# Phage therapy of perinephric abscess in kidney transplantation recipients caused by drug‐resistant *Pseudomonas aeruginosa*

**DOI:** 10.1002/mlf2.70042

**Published:** 2025-12-25

**Authors:** Jiazhen Liu, Shuguang Lu, Juan Bao, Jing Wang, Yali Gong, Bo He, Yuhao Zhu, Zhuojun Zhong, Shiru Li, Linlin Li, Na Li, Mengjun Cheng, Ming Li, Mengyu Shen, Nannan Wu, Tongyu Zhu, Shuai Le

**Affiliations:** ^1^ Department of Microbiology, Key Laboratory of Microbial Engineering Under the Educational Committee in Chongqing, College of Basic Medical Sciences Army Medical University Chongqing China; ^2^ Shanghai Institute of Phage, Shanghai Public Health Clinical Center Fudan University Shanghai China; ^3^ Burn Institute, Southwest Hospital Army Medical University Chongqing China; ^4^ Department of Infectious Diseases Zhongshan Hospital, Fudan University Shanghai China; ^5^ Shanghai Institute of Infectious Disease and Biosecurity, Shanghai Public Health Clinical Center Fudan University Shanghai China; ^6^ Fudan University Phage Institute Zhongshan Hospital Fudan University Shanghai China; ^7^ CAS Key Laboratory of Microbial Physiological and Metabolic Engineering, State Key Laboratory of Microbial Resources, Institute of Microbiology Chinese Academy of Sciences Beijing China; ^8^ Basic Medical Laboratory General Hospital of Central Theater Command Wuhan China; ^9^ CreatiPhage Biotechnology, Co. Ltd. Shanghai China; ^10^ Shanghai Medical College, Fudan University Shanghai China; ^11^ Shanghai Key Laboratory of Organ Transplantation, Department of Kidney Transplantation Zhongshan Hospital, Fudan University Shanghai China

## Abstract

Perinephric abscess, a common complication after kidney transplantation, often results from drug‐resistant bacterial infections and is notoriously difficult to treat. Phage therapy has emerged as a promising alternative for such resistant infections. Here, we present two cases of perinephric abscesses in kidney transplant recipients (KTRs) treated with phage therapy. Our findings highlight the need for personalized treatment plans and timely intervention with phage therapy to improve patient outcomes. Future research should focus on overcoming barriers like biofilms to make this treatment more effective. Ultimately, phage therapy could lead to better survival rates and improved quality of life for transplant patients facing severe infections. This study is a step forward in the fight against superbugs, offering a potential alternative when antibiotics fail.

## INTRODUCTION

Perinephric abscess is a widely recognized postoperative infectious complication that may occur at any time after kidney transplantation, presenting significant treatment challenges^1^, ^2^. In severe cases, it can progress to sepsis and may even necessitate allograft nephrectomy^3^. The predominant pathogens associated with perinephric abscess include *Staphylococcus* spp., *Enterobacteriaceae*, and *Pseudomonas aeruginosa* ^4^, ^5^. *P. aeruginosa* proves particularly difficult to eradicate due to its high abundance, rapid growth rate, highly adaptable genome, biofilm‐forming capability, and especially its intrinsic multidrug resistance^6^, ^7^, ^8^, ^9^.

Recently, interest has resurged in utilizing phages as targeted therapeutic agents against multidrug‐resistant bacterial infections^10^, ^11^, ^12^. Phages can rapidly eliminate *P. aeruginosa* while simultaneously replicating new phage particles and demonstrating potent biofilm‐disrupting activity, particularly when combined with antibiotics^13^, ^14^. Multiple case reports have demonstrated both the safety and efficacy of phage therapy, whether administered locally or systemically. Successful applications of phage therapy have been documented for *P. aeruginosa* infections in various sites, including the respiratory tract, prosthetic knees, aortic grafts, and bone joints^15^, ^16^. Notably, phage therapy has shown significant promise for immunosuppressed lung and liver transplant recipients infected with *P. aeruginosa* ^17^, ^18^. This potential is exemplified by the successful case of a toddler who underwent liver transplantation following bacteriophage–antibiotic combination therapy, underscoring its effectiveness against persistent infections in complex clinical scenarios^19^.

In this study, two kidney transplant recipients with perinephric abscesses received treatment with personalized phage cocktails. Patient 1, presenting with an acute infection, achieved complete recovery. However, in Patient 2, with a chronic infection, the *P. aeruginosa* infection was not fully eliminated due to established biofilm formation and complex sinus tract anatomy. These two cases demonstrate both the safety and efficacy of phage therapy for perinephric abscesses in kidney transplant recipients (KTRs). Our findings suggest that phage therapy should be initiated promptly, before stable biofilm formation^20^, ^21^, ^22^, to maximize bacterial eradication potential.

## RESULTS

### Phage therapy for acute perinephric abscess in a kidney transplant recipient

**Figure 1:**
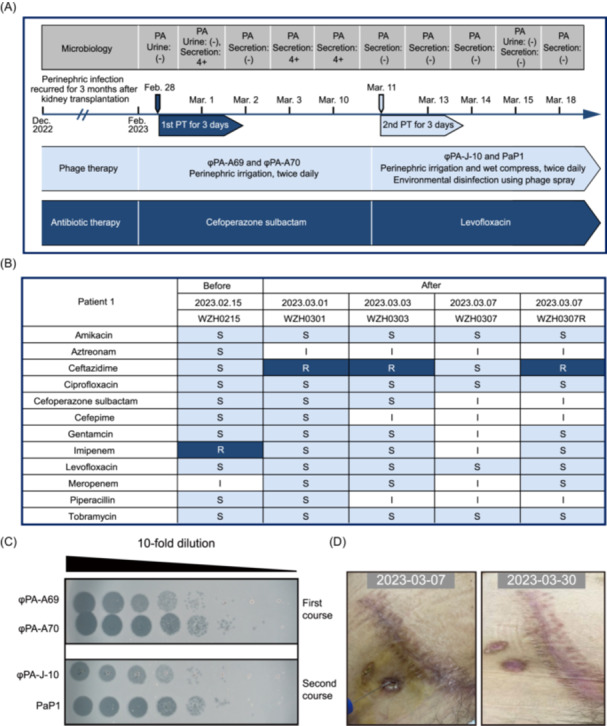
Process and efficacy of phage therapy for Patient 1. (A) Timeline of microbiological results, phage therapy, and antibiotic therapy for Patient 1. Two courses of phage therapy were administered with two distinct 2‐phage cocktails, each lasting for 3 days. PT, phage therapy. (B) A test on the antibiotic sensitivity of *Pseudomonas aeruginosa* isolates carried out before and after phage therapy. The light blue box represents susceptibility, the deep blue box indicates resistance, and the white box denotes intermediate resistance. S indicates antibiotic sensitivity, I indicates intermediate antibiotic resistance, and R indicates antibiotic resistance. (C) The efficiency of plating (EOP) assay used to detect phage sensitivity to *P. aeruginosa* strains before each course of treatment. (D) Two local applications of phage cocktails effectively eradicated abdominal wound infection. PA, *Pseudomonas aeruginosa.*

Patient 1 was a 23‐year‐old male who received a kidney transplant in December 2022. Postoperatively, he was prescribed standard immunosuppressive therapy consisting of mycophenolate enteric‐coated tablets, prednisone, and abatacept. One month post‐transplantation, he developed a perinephric abscess in the grafted kidney, accompanied by asymptomatic bacteriuria caused by antibiotic‐resistant *P. aeruginosa*. Notably, the patient had neither a ureteral stent nor a urinary catheter in place; instead, a perinephric catheter was inserted for abscess drainage. Despite receiving multiple antibiotic regimens (including linezolid, vancomycin, and piperacillin‐tazobactam) for 2 weeks at a local hospital, the treatment failed to resolve the infection, with persistent purulent discharge from the sinus tract. In February 2023, the patient was referred to the Shanghai Public Health Clinical Center for phage therapy (Figure 1A,B).

Patient 1 received treatment with a two‐phage cocktail consisting of φPA‐A69 and φPA‐A70 (each at 10⁸ plaque‐forming units [PFU]/ml). This phage combination demonstrated effective lytic activity against the patient's *P. aeruginosa* strain WZH0215 (Figure 1C). For administration, 10 ml of the phage cocktail (φPA‐A69 + φPA‐A70) was diluted in 100 ml of Lactated Ringer's solution. The resulting mixture was used for perinephric irrigation through the drainage tube twice daily (with 12‐h intervals between administrations) over a 3‐day treatment course. Concurrently, intravenous cefoperazone‐sulbactam was administered as the antibiotic component, selected based on antimicrobial susceptibility testing results (Figure 1B).

Bacterial strains WZH0215, WZH0301, WZH0303, WZH0307, and WZH0307R were isolated before and after phage therapy. Notably, these strains displayed varying antibiotic resistance profiles, showing either sensitivity or resistance to ceftazidime. However, their phage sensitivity remained consistent, with all isolates maintaining susceptibility to both φPA‐A69 and φPA‐A70 (Figure 1C). To expedite treatment and avoid delays associated with custom phage cocktail production, we promptly selected two alternative phage preparations from our existing stock.

Subsequently, a second phage cocktail (φPA‐J‐10 and PaP1, each at 10⁸ PFU/ml) was administered via perinephric irrigation for 3 days. Following each irrigation session, the same phage solution was applied as a 30‐min wet compress. Based on antibiotic susceptibility testing results (Figure 1B), the adjunct antibiotic therapy was changed from cefoperazone‐sulbactam to levofloxacin.

After completing this second course of phage therapy, the patient showed excellent tolerance and was discharged on March 13, 2023. At discharge, both wound and urine cultures showed no bacterial growth. The drainage tube was successfully removed, with complete sinus tract closure observed shortly thereafter (Figure 1D). During the 6‐month follow‐up period, no *P. aeruginosa* recurrence was detected. These findings suggest that early phage intervention should be considered for antibiotic‐resistant perinephric abscesses in kidney transplant recipients. Additionally, ensuring sufficient treatment duration appears to be critical for achieving complete pathogen eradication.

### Phage therapy for chronic perinephric abscess in a kidney transplant recipient

**Figure 2:**
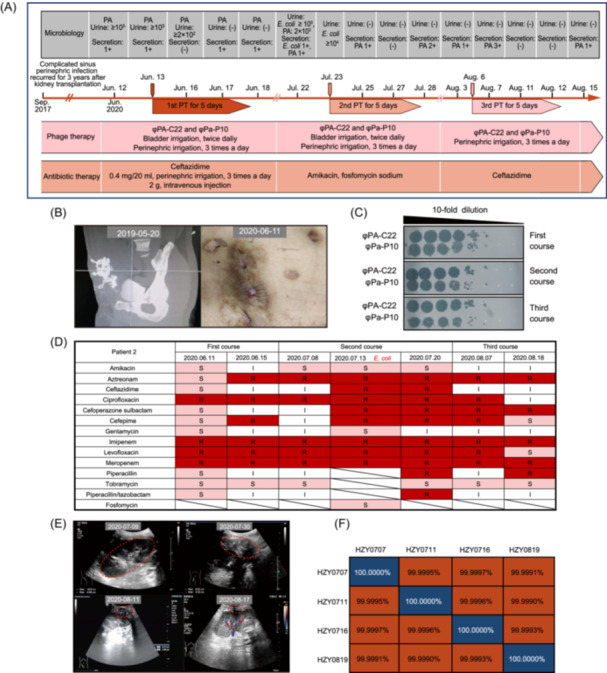
Process and efficacy of phage therapy for Patient 2. (A) Timeline of microbiological results, phage therapy, and antibiotic therapy for Patient 2. Three courses of phage therapy were administered with the identical 2‐phage cocktail, each lasting 5 days. (B) The sinus tract beneath the wound observed via a computerized tomography (CT) scan in 2019, and the image of the abdominal wound before PT. (C) The EOP assay used to determine the phage sensitivity to strains before each course of treatment. (D) A test on the antibiotic sensitivity of wound isolates conducted before and after three courses of phage therapy. The pink box indicates susceptibility, the deep red represents resistance, the white indicates intermediate resistance, and the slash denotes untested. (E) Four ultrasound examinations of the transplanted kidney performed on July 9, July 30, August 11, and August 17. The results were presented as follows: a 72 × 15 mm low‐echo nonhomogeneous area at the lower pole of the transplanted kidney on July 9, 63 × 33 mm on July 30, 42 × 23 mm on August 11, and 21 × 10 mm on August 17. The low‐echo nonhomogeneous areas were delineated by red dotted lines. (F) A heatmap of the genome pairing similarity of four *P. aeruginosa* strains based on average nucleotide identity (ANI) values indicated that these strains originated from the same progenitor strain.

Patient 2 was a 31‐year‐old male who received a kidney transplant in September 2017. He subsequently developed postoperative perinephric hemorrhage. During two hematoma evacuation procedures, surgeons placed a significant amount of hemostatic gel in the perinephric space. In February 2018, the patient underwent renal pelvis–ureter–bladder reimplantation with bladder muscular flap ureteroplasty, during which a ureteral stent was placed and a nephrostomy tube was maintained. Following this procedure, the patient developed a perinephric infection caused by pan‐drug‐resistant *P. aeruginosa* (PDR‐PA) (Figure 2A), which led to the formation of an abdominal fistula originating from the nephrostomy tube site, with persistent purulent drainage (Figure 2B). Although intermittent antibiotic therapy was administered to control the infection, complete eradication was not achieved. From December 2018 onward, the patient required multiple hospital admissions for febrile episodes and anti‐infective treatment. During these hospitalizations, PDR‐PA was consistently isolated from both wound exudate and urine specimens, with no lasting response to antibiotic therapy. In June 2020, the patient was referred to the Shanghai Public Health Clinical Center for phage therapy.

Patient 2 received treatment with a phage cocktail containing two phages (φPA‐C22 and φPa‐P10), each at a titer of 10⁹ PFU/ml (Figure 2C). The therapeutic solution was prepared by mixing 20 ml of each phage with lactated Ringer's solution to yield a final volume of 400 ml. The treatment regimen included both perinephric and bladder irrigation approaches. In the first round of treatment, 150 ml of the phage cocktail dilutions were utilized for bladder irrigation twice daily for 5 days, with a 12‐h interval between each irrigation. This was combined with 2 g of ceftazidime administered intravenously every 8 h.

At the end of the first treatment round, *P. aeruginosa* was no longer detected in the urine but persisted in wound secretions. Given the continued susceptibility of the isolates to the original phage cocktail, an identical second round of phage therapy was administered. However, before initiating the second round, a drug‐resistant *Escherichia coli* infection emerged. Consequently, ceftazidime was discontinued and replaced with combination therapy using amikacin and fosfomycin sodium (Figure 2D), which successfully eradicated the *E. coli*. While *P. aeruginosa* remained absent from the urine, it persisted in secretions. The third treatment round focused exclusively on the perinephric infection, as bladder irrigation was discontinued following confirmed bacterial clearance in the urine. Despite this intervention, *P. aeruginosa* remained detectable in secretions 5 days posttreatment (Figure 2A).

After three rounds of phage therapy, the pathogenic bacterial load was effectively controlled, with the inflammatory area significantly reduced from 72 × 15 mm to 21 × 10 mm, as confirmed by ultrasound examination (Figure 2E). However, complete eradication of *P. aeruginosa* was not achieved. Due to personal circumstances, the patient elected for hospital discharge. During 6 months of follow‐up, he maintained a stable clinical status and normal quality of life through routine wound care management.

### Comparative genomics of *P. aeruginosa* before and after phage therapy

Genomic analysis was performed on *P. aeruginosa* isolates HZY0707, HZY0711, HZY0716, and HZY0819 collected during and after phage therapy of Patient 2. Whole‐genome sequencing revealed an average nucleotide identity (ANI) exceeding 99.999%, confirming that these strains were clonal derivatives of the same ancestral strain (Figure 2F). When compared to the reference strain HZY0707, the other three isolates showed only 6–10 single‐nucleotide polymorphisms (SNPs) or indels (Table 1). Importantly, none of these genetic variations affected genes related to lipopolysaccharide (LPS) or pili biosynthesis—the primary phage receptors in *P. aeruginosa*. Consistent with this finding, all strains remained susceptible to the original phage cocktail (φPA‐C22 and φPa‐P10) (Figure 2C).

**Table 1:** SNPs and genomic deletions in *Pseudomonas aeruginosa* isolated from Patient 2.

Patient 2's strain	Position	Mutation	Annotation	Gene		Description		Function		Isolation date	
HZY_0707	Reference strain									2020.07.07 (during PT)	
HZY_0711	350,712	166,524	Intergenic (−92/−284)	(–)		(–)		(–)		2020.07.11 (during PT)	
	19,453	Δ12 bp	Coding (29–40/408 nt)	*ctg00008_0357*		Hypothetical protein		Hypothetical protein			
	1778	Δ2 bp	Coding (253–254/915 nt)	*nodD2_2*		Nodulation protein D 2		Regulate the expression of the nodABCFE genes			
	2123	C → T	P200S (CCC → TCC)	*nodD2_2*		Nodulation protein D 2		Regulate the expression of the nodABCFE genes			
	2995	G → A	A109T (GCC → ACC)	*bepF_2*		Efflux pump periplasmic linker BepF		Acra/E‐related components of drug efflux transporter			
	244,196	A → T	Intergenic (−27/ + 87)	(–)		(–)		(–)			
	130,673	+GCC	Coding (136/1077 nt)	*ssuD_3*		Alkanesulfonate monooxygenase		Oxidoreductase activity, acting on paired donors, with incorporation or reduction of molecular oxygen			
	156,584	C → T	G287S (GGC → AGC)	*leuO*		HTH‐type transcriptional regulator LeuO		Involving in the catabolism of nitroaromatic/naphthalene compounds and the catabolism of related regulators			
	161,391	A → G	E538E (GAA → GAG)	*betA_1*		Oxygen‐dependent choline dehydrogenase		Amino acid biosynthesis and metabolism			
	99,725	A → C	V160G (GTG → GGG)	*macB_3*		Macrolide export ATP‐binding/permease protein MacB		ABC transporters			
HZY_0716	259,081	(T)8→(T)7	Intergenic (+42/+29)	(–)		(–)		(–)		2020.07.16 (during PT)	
	1680	Δ15 bp	Coding (155‐169/915 nt)	*nodD2_2*		Nodulation protein D 2		Regulate the expression of the nodABCFE genes			
	2123	C → T	P200S (CCC → TCC)	*nodD2_2*		Nodulation protein D 2		Regulate the expression of the nodABCFE genes			
	244,196	A → T	Intergenic (−27/+87)	(–)		(–)		(–)			
	130,673	+GCC	Coding (136/1077 nt)	*ssuD_3*		Alkanesulfonate monooxygenase		Oxidoreductase activity, acting on paired donors, with incorporation or reduction of molecular oxygen			
	161,391	A → G	E538E (GAA → GAG)	*betA_1*		Oxygen‐dependent choline dehydrogenase		Amino acid biosynthesis and metabolism			
HZY_0819	166,524	G → A	R381C (CGC → TGC)	*sir_1*		Sulfite reductase (ferredoxin)		tRNA wobble position uridine thiolation		2020.08.19 (during PT)	
	439,464	Δ1 bp	Coding (2413/2937 nt)	*btuB_5*		Vitamin B12 transporter BtuB		Cobalamin transport			
	1680	Δ15 bp	Coding (155–169/915 nt)	*nodD2_2*		Nodulation protein D 2		Regulate the expression of the nodABCFE genes			
	2123	C → T	P200S (CCC → TCC)	*nodD2_2*		Nodulation protein D 2		Regulate the expression of the nodABCFE genes			
	244,196	A → T	Intergenic (−27/+87)	(–)		(–)		(–)			
	130,673	+GCC	Coding (136/1077 nt)	*ssuD_3*		Alkanesulfonate monooxygenase		Oxidoreductase activity, acting on paired donors, with incorporation or reduction of molecular oxygen			
	161,391	A → G	E538E (GAA → GAG)	*betA_1*		Oxygen‐dependent choline dehydrogenase		Amino acid biosynthesis and metabolism			
	113,798	C → T	W439* (TGG → TGA)	*btuB_10*		Vitamin B12 transporter BtuB		Cobalamin transport			
	150,340	C → T	G180S (GGC → AGC)	*pstS*		Phosphate‐binding protein PstS		Single‐species biofilm formation			

PT, phage therapy.

These results demonstrate that while the bacterial strains underwent continuous evolution during chronic infection, no phage‐resistant mutants emerged throughout the treatment course. The persistence of *P. aeruginosa* infection likely resulted from either biofilm formation or anatomical barriers (complex sinus tracts) that physically prevented phage–bacteria interactions, rather than genetic resistance mechanisms.

## DISCUSSION

Kidney transplantation has become increasingly common due to advances in surgical techniques, immunosuppression protocols, and antimicrobial prophylaxis, leading to improved allograft outcomes^2^. However, infections remain a frequent complication and major limiting factor in kidney transplantation^23^. The period of greatest immunosuppression occurs during the first 3–6 months post‐transplantation, coinciding with the highest infection risk. Perinephric abscesses represent a significant complication, with risk factors including postoperative lymphocele, urine leakage, hematoma, and reoperation for bleeding^24^. While phage therapy shows promise for treating antibiotic‐resistant *P. aeruginosa* infections^25^, its application for perinephric abscesses in KTRs remains limited. We present two relevant cases here.

Patient 1 received two courses of phage cocktail therapy combined with targeted antibiotics; after the first course of phage therapy, the stockpiles of φPA‐A69 and φPA‐A70 have been exhausted, to shorten the treatment waiting period, an equivalent sensitive phage combination φPA‐J‐10 and PaP1 was substituted for the second course of treatment. Based on antimicrobial susceptibility testing before the second phage course, antibiotics were switched from cefoperazone‐sulbactam to levofloxacin, resulting in clinical improvement. Both wound secretions and urine became culture‐negative, with complete fistula closure. Notably, although the initial 3‐day phage regimen proved insufficient, an additional 3‐day course successfully eradicated all bacterial strains. This suggests that phage therapy should be continued until complete pathogen clearance is achieved, provided that no resistance emerges.

Patient 2 underwent three phage therapy courses with adjunct antibiotics. Since the cocktail (φPA‐C22 and φPa‐P10) was sensitive to the bacteria isolated before the three courses of phage treatment, there was no change in the phage cocktail combination. Although perinephric *P. aeruginosa* persisted, urinary tract infection resolved, with reduced wound drainage and sinus tract narrowing. Chronic, drug‐resistant *P. aeruginosa* infections within complex sinus tracts proved particularly refractory to eradication. Sensitivity testing confirmed that all isolates remained phage‐susceptible despite accumulating genomic variations (SNPs/indels). The treatment failure likely resulted from anatomical barriers preventing phage penetration rather than bacterial resistance.

Previous research confirms that complex anatomical structures like sinus tracts can limit phage distribution and efficacy^26^, ^27^, ^28^, underscoring the need to overcome these barriers. Innovative delivery methods^29^, ^30^, ^31^ and combined surgical approaches (e.g., sinus resection with drainage) may improve outcomes^32^. For Patient 2, open abdominal debridement was deemed too risky, given the post‐transplant context.

Bladder irrigation successfully cleared *P. aeruginosa* where direct phage exposure was possible, contrasting with the protected perinephric environment. This suggests that for deep‐seated infections, combing surgical drainage/debridement with phage therapy warrants consideration and further study.

Overall, these two cases provide important insights for personalized phage therapy in KTRs with perinephric abscesses. They highlight the need for both basic research and clinical investigations to optimize phage therapy efficacy, particularly for anatomically complex infections.

## MATERIALS AND METHODS

### *P. aeruginosa* clinical isolates and antibiotic susceptibility test (AST)

Clinical strains were isolated from the patient's sample and inoculated onto blood agar plates. Subsequently, they were identified by matrix‐assisted laser desorption/ionization time‐of‐flight mass spectrometry (Vitek MS, bioMérieux), with the identification compared against the IVD 3.0 database. The antimicrobial susceptibility was evaluated using the Vitek 2 system (bioMérieux) following the manufacturer's guidelines. Specifically, susceptibility cards designed for Gram‐negative bacteria (Vitek AST‐N335) were utilized, and all experiments were conducted in triplicate.

### Preparation of phage solutions

The phage agent was prepared following the method described previously^33^. Active phages against the patient's *P. aeruginosa* isolates were selected from the Bacteriophage Library (Shanghai Institute of Phage) using the spot test method and the double agar overlay method. The susceptibilities of the target bacteria to phages were determined by the efficiency of plating (EOP) assay as described previously^34^.

The phages were cultivated in the Luria–Bertani medium. Subsequently, they were subjected to chloroform sterilization and centrifuged at 12,000 rpm for 10 min at 4°C. The resulting supernatant was filtered through a 0.22‐micron membrane and further purified using a CIM® Anion‐exchange column QA (BIA Separations). Subsequently, the EOP assay was conducted to determine the phage titer against the specific strain^35^. Briefly, the phage solution was serially diluted 10‐fold, and then 2 µl of the diluted solution was placed onto an agar plate seeded with bacteria in the logarithmic growth phase. The plates were then incubated at 37°C overnight. The candidate phages were selected due to their strong antibacterial activity, which was manifested by the formation of large and clear plaques. All phage solutions with a concentration of 10^8^ PFU/ml were aliquoted and packaged following good manufacturing practice (GMP) standards at the GMP Workshop of Zhongshan Hospital, Fudan University in Shanghai, China.

### Genome sequencing and analysis of *P. aeruginosa* clinical strains

The genomes of all the isolated *P. aeruginosa* clinical strains were sequenced via Illumina Miseq sequencing platforms. Once the sequencing process was accomplished, mutations were identified in clean reads by using Breseq with the specified reference genome^36^. Subsequently, we calculated the ANI values based on the whole‐genome FASTA sequences of the strains^37^. Then, we calculated the ANI values of all four strains isolated from Patient 2 and performed pairwise comparisons among all the strains. A matrix file was generated as an output by FastANI and was subsequently imported into Excel for plotting purposes.

## AUTHOR CONTRIBUTIONS

**Liu Jiazhen:** Data curation; visualization; writing—original draft. **Lu shuguang:** Software; writing—original draft; writing—review & editing. **Bao Juan:** Data curation; resources; writing—original draft. **Wang Jing:** Investigation; supervision. **Gong Yali:** Investigation; methodology. **He Bo:** Formal analysis; investigation; supervision. **Zhu Yuhao:** Data curation; software. **Zhong Zhuojun:** Data curation; software; visualization. **Li Shiru:** Software; validation. **Li Linlin:** Methodology; project administration; resources. **Li Na:** Investigation; resources; software. **Cheng Mengjun:** Formal analysis; supervision; validation. **Li Ming:** Resources. **Shen Mengyu:** Funding acquisition; resources. **Wu Nannan:** Conceptualization; formal analysis; project administration; resources. **Zhu Tongyu:** Conceptualization. **Le Shuai:** Conceptualization; funding acquisition; resources; writing—review & editing.

## ETHICS STATEMENT

This study was approved by the Ethics Committee of Shanghai Public Health Clinical Center (2020‐S15701). The patients provided informed consent for phage therapy and the utilization of samples and data in research and publication.

## CONFLICT OF INTERESTS

The authors declare no conflict of interests.

## Data Availability

Bacterial genomes were deposited in NCBI (BioProject ID: PRJNA1193305‐PRJNA1193308).
